# Multifunctional Biological Activity Assessment of Plant-Derived Nanovesicles from Arugula Leaves: In Vitro and In Vivo Studies

**DOI:** 10.3390/antiox14121421

**Published:** 2025-11-27

**Authors:** Lorenza d’Adduzio, Melissa Fanzaga, Davide Marangon, Antonio Carrillo-Vico, Ivan Cruz-Chamorro, Carlotta Bollati, Davide Lecca, Carmen Lammi

**Affiliations:** 1 Department of Pharmaceutical Sciences, University of Milan, 20133 Milan, Italy; 2 Departamento de Bioquímica Médica y Biología Molecular e Inmunología, Facultad de Medicina, Universidad de Sevilla, 41009 Seville, Spain; 3 Instituto de Biomedicina de Sevilla, IBiS (Universidad de Sevilla, HUVR, Junta de Andalucía, CSIC), 41013 Seville, Spain; 4 Facultad de Enfermería, Universidad de Castilla-La Mancha, 02071 Albacete, Spain

**Keywords:** bioactive plant nanovesicles, antioxidant activity, cholesterol lowering effect, multifunctional activity

## Abstract

Plant-derived vesicles (PDVs) represent an emerging class of naturally bioformulated nanocarriers with potential nutraceutical and therapeutic applications. In this study, the multifunctional biological activity of PDVs obtained from *Eruca sativa* leaves (arugula leaf vesicles, ALVs) was investigated both in vitro and in vivo. In differentiated Caco-2 and HepG2 cells, ALVs exhibited significant antioxidant activity, being rich in polyphenols and organic acids, by reducing intracellular reactive oxygen species (ROS) and modulating key metabolic regulators. ALVs upregulated SREBP-2, LDLR, and phosphorylated AMPK and Akt, leading to enhanced LDL and glucose uptake, while downregulating FASN and PPAR-γ, thereby reducing lipid accumulation. In mice fed a high-fat and high-fructose (HFHF) diet, ALV supplementation improved glucose tolerance and decreased total cholesterol, LDL, and hepatic injury biomarkers (ALT, AST, and LDH) without inducing toxicity. These findings demonstrate that ALVs exert hypocholesterolemic, hypoglycemic, and lipid-lowering effects through coordinated modulation of AMPK/Akt pathways. Overall, ALVs emerge as safe, multifunctional nanovesicles capable of counteracting oxidative stress and metabolic dysfunction, highlighting their potential as innovative bioactive ingredients for functional foods or nutraceutical formulations targeting metabolic syndrome.

## 1. Introduction

Plant-derived vesicles (PDVs) are circular membrane-bound structures that carry genetic material (miRNAs, DNA, and RNA) [1], lipids, proteins, and metabolites [2,3] that reflect the phytochemical profile of the plant matrix they originate from. Recently, some heterogenous studies highlighted that PDVs exert intestinal anti-inflammatory and antioxidant activities in some cellular and animal models [4,5,6,7]. Although vesicles derived from grapes, grapefruit, and ginger have been shown to support intestinal functionality and anti-inflammatory activity [8,9], their structural properties, low human immunogenicity, stability in the gastrointestinal tract, and ability to cross the blood–brain barrier warrant further characterization [10]. Differently from vesicles sourced from animal cells (e.g., exosomes and microvesicles), the exploration of PDVs represents a nascent field with numerous unresolved aspects, as evidenced by a thorough review of the literature [11,12]. One recurring issue is the oversight of the distinction between apoplastic exosome-like native vesicles, akin to those found in mammals, which are naturally produced by plants in response to stress stimuli, and PDVs resulting from tissue processing, that can be artificially generated during proper plant tissue processing and lack relevance to plant biology [13]. Indeed, while the production of apoplastic vesicles occurs in limited quantities, posing challenges for their isolation and large-scale production [14], the natural abundance of PDVs from plants offers a sustainable, scalable alternative. Undoubtedly, the unique physico-chemical and structural characteristics of PDVs make them naturally bioformulated nanocarriers of bioactives that have gained research interest in recent years, with promising applications in many fields, such as pharmaceutical, nutraceutical, and cosmetic ones. However, the most employed techniques for obtaining and isolating PDVs involve the ultracentrifugation method, which leads to PDV aggregate formation and/or membrane integrity loss, impairing PDV functionality and strongly impacting the yield of the method, thus compromising its application and scale-up at an industrial level.

Recently, to fill these gaps and advance the research field on PDVs, we developed a standardized and adaptable method—International Publication Number WO 2024/223549 A1 [15], licensed to Plantech s.r.l. (https://www.plantechlab.com/home-page/, accessed on 20 October 2025)—to obtain, isolate, and store PDVs, preserving their morphology, integrity, physicochemical characteristics, and bioactive contents. By applying the production and storage method on *Eruca sativa* leaves, a sample containing 67 (±0.2) × 10^6^ arugula leaf nanovesicles (ALVs)/mg that carry plant miRNAs and secondary metabolites, mainly polyphenols and carboxylic acids, was obtained. ALVs were demonstrated to be safe and bioavailable in a dose-dependent manner in vitro on differentiated Caco-2 cells [16]. Brassica vegetables, including broccoli, arugula, Brussels sprouts, cabbage, and cauliflower, are rich in nutrients, such as carbohydrates, proteins, fiber, vitamins (C, K, E, and B9), and essential minerals like iron, selenium, calcium, copper, and zinc [17]. It is well known that they also contain various functional metabolites, including glucosinolates, flavonoids, carotenoids, antioxidant enzymes, and terpenes, which contribute to their beneficial health effects [18,19]. These bioactive compounds exhibit strong anti-inflammatory and antioxidant properties in the liver, potentially influencing lipid production and metabolism [20]. Taking these aspects into account, and considering the complex and heterogeneous ALV composition, the aim of the present study was to investigate the ALV biological properties both in vitro and in vivo. More in detail, the mechanisms of action through which ALVs modulate the cholesterol, glucose, and lipid metabolism on HepG2 cells were evaluated by using a combination of molecular and functional techniques. Lastly, the *proof of concept* of their safety and multifunctional hypolipemic and hypoglycemic activities was confirmed in a mouse model, in which the metabolic syndrome was induced by a high-fat and high-fructose (HFHF) diet. Overall, our results cross the boundaries between food science, food biotechnology, pharmaceutical science, and nanotechnology, opening a new multidisciplinary area of research, and suggesting that ALVs could have a great potential to become new food bioactive ingredients to be exploited to produce innovative dietary supplements or excellent natural bio-engineered carriers for the delivery of bioactive compounds in target cells.

## 2. Materials and Methods

### 2.1. Chemicals

All chemicals and reagents were of analytical grade and from commercial sources. The 3-(4,5-dimethylthiazol-2-yl)-2,5-diphenyltetrazolium bromide (MTT) and Oil Red O solution were from Sigma-Aldrich (St. Louis, MO, USA). Bovine serum albumin (BSA) and Bradford reagent were purchased from Bio-Rad (Hercules, CA, USA). Dulbecco’s modified Eagle medium (DMEM), Medium 199, fetal bovine serum (FBS), L-glutamine, phosphate-buffered saline (PBS), penicillin/streptomycin, and 96-well plates were from Euroclone (Milan, Italy). Polycarbonate Centrifuge Tubes were from Beckman Coulter, Optima TL, Brea, CA, USA.

### 2.2. Obtainment, Purification, and Stabilization of ALVs

ALVs from *E. sativa* were produced following the patented method for the production, purification, and stabilization of plant-derived nanovesicles [15]. Briefly, commercially available ready-to-eat arugula leaves were purchased from a local supermarket in Milan (Italy) and pooled to minimize variability associated with differences batch and supplier, thereby ensuring a more representative and standardized starting material for our analyses. The procedure comprises the following steps: homogenization of the vegetable matrix using a blender to obtain a homogenate; separation (by sieving) of the juice from the solid residue present in the homogenate; isolation and purification of the resulting nanovesicles through ultrafiltration with two molecular weight cut-offs (100 and 10 kDa), coupled with size-exclusion chromatography using an Izon qEV Original (Christchurch, New Zealand) 35 nm column, from which specific eluted fractions were collected; stabilization of the nanovesicles and nanoparticles by spray-drying. Further details are provided in our patent [15].

### 2.3. Morphological Analysis by Cryogenic Electron Microscopy (Cryo-EM)

Spray-dried ALVs were resuspended in water under saturated conditions. Sample vitrification was performed using a Mark IV Vitrobot (Thermo Fisher Scientific, Waltham, MA, USA). A volume of 3 μL was applied onto Quantifoil R 2/1 Cu 300-mesh and Lacey grids, both previously glow-discharged at 30 mA for 30 s using a GloQube system (Quorum Technologies, Laughton, UK). Immediately after sample application, the grids were blotted in a controlled chamber set at 4 °C and 100% humidity, and subsequently plunge-frozen in liquid ethane. The vitrified grids were transferred to a Talos Arctica transmission electron microscope (Thermo Fisher Scientific) operating at 200 kV and equipped with a Ceta 16M detector (Thermo Fisher Scientific, Waltham, MA, USA). Images were collected at nominal magnifications ranging from 22k× to 45k×, corresponding to pixel sizes of 4.66 Å/pixel and 2.29 Å/pixel, respectively.

### 2.4. ALV Chemical Composition Analysis: Secondary Metabolite Determination by HR-HPLC-MS/MS

Polyphenol extraction was performed in triplicate, and the entire procedure was carried out in darkness. ALV powder was homogenized in acidified methanol (0.1% *v*/*v* HCl) at 4% *w*/*v*. The mixture was then placed in an ultrasonic bath for 15 min and centrifuged at 12,500 rpm for 5 min. The pellet was discarded, and the supernatant was stored at –80 °C until analysis. Samples were analyzed at UNITECH OMICs (University of Milan, Italy) using the ExionLC™ AD system (SCIEX, Framingham, MA, USA) coupled to the ZenoTOF 7600 system (SCIEX) equipped with a Turbo V™ Ion Source and ESI probe (SCIEX, Singapore). Further information is available in Appendix A.

### 2.5. Cell Culture Conditions

HepG2 (HB-8065 and ATCC; LGC Standards, Milan, Italy) and Caco-2 cells (INSERM, Paris, France) were maintained in high-glucose DMEM containing stable L-glutamine and supplemented with 10% FBS, 100 U/mL penicillin, and 100 μg/mL streptomycin (complete growth medium) at 37 °C in a humidified 5% CO_2_ atmosphere. Caco-2 cells were routinely passaged at ~50% confluence [21]. HepG2 cells were used for no more than 20 passages after thawing, as additional passages can alter cell morphology and characteristics and compromise assay outcomes.

### 2.6. Trans-Epithelial Transport: Caco-2 Cell Culture and Differentiation

Human intestinal Caco-2 cells obtained from INSERM (Paris, France) were cultured according to a published protocol [21]. For differentiation, cells were seeded onto polycarbonate filters (Transwell, Corning Inc., Lowell, MA, USA; 12 mm diameter and 0.4 μm pore size) at 3.5 × 10^5^ cells/cm^2^ in complete medium, with 10% FBS added to both apical (AP) and basolateral (BL) compartments for 2 days to establish a confluent monolayer. From day 3 after seeding, cells were transferred to FBS-free medium in both AP and BL compartments, and allowed to differentiate for 18–21 days, with medium changes three times per week [22].

### 2.7. 3-(4,5-Dimethylthiazol-2-yl)-2,5-diphenyltetrazolium Bromide (MTT) Assay

The MTT assay was performed on human intestinal Caco-2 cells. Briefly, 3 × 10^4^ cells/well were seeded into 96-well plates and, after 24 h, treated with ALVs (0.01–1 mg/mL) or vehicle in complete growth medium for 48 h at 37 °C and 5% CO_2_. The treatment was then removed, and 100 μL/well of filtered 0.5 mg/mL MTT solution was added. After 2 h at 37 °C under 5% CO_2_, the MTT solution was aspirated and 100 μL/well of lysis buffer (8 mM HCl + 0.5% NP-40 in DMSO) was added. Following 10 min of gentle shaking, absorbance at 575 nm was measured on a Synergy H1 fluorescence plate reader (Biotek, Bad Friedrichshall, Germany).

### 2.8. Fluorometric Intracellular ROS Assay on Intestinal Caco-2 Cells

To evaluate the antioxidant properties of ALVs in Caco-2 cells, an intracellular ROS assay was performed using a standard protocol with minor modifications [23]. In brief, Caco-2 and HepG2 cells (5 × 10^4^) were seeded and, the following day, incubated with ALVs (0.1 and 0.5 mg/mL) for 1 h in the dark. ROS formation was induced with H_2_O_2_ (1 mM) for 30 min at 37 °C in the dark. Fluorescence (ex/em 490/525 nm) was recorded using a Synergy H1 microplate reader (Biotek Instruments, Winooski, VT, USA).

### 2.9. Caco-2 Cells Monolayer Integrity Evaluation

The transepithelial electrical resistance (TEER) of differentiated Caco-2 monolayers was measured at 37 °C using a Millicell voltmeter (Millipore Co., Billerica, MA, USA) immediately before and at the end of transport experiments. Only filters showing TEER values were included in the ALV transport analysis.

### 2.10. Caco-2/HepG2 Co-Culture Development and ALVs Treatment

ALV treatments were carried out on 21-day-differentiated intestinal Caco-2 cells co-cultured with HepG2 cells seeded on the bottom of the culture plate. For co-culture, Caco-2 inserts were transferred into multiwell plates containing confluent HepG2 cells. Prior to ALV exposure, differentiated Caco-2 cells were washed twice with 500 μL of PBS containing 1 mM Ca^2+^ and 1 mM Mg^2+^. The AP compartment received 67 × 10^6^ ALVs/mL (equivalent to 1 mg/mL ALVs), while the BL compartment contained 700 μL of complete medium with 10% FBS. After 24 h of co-culture, HepG2 cells were harvested for downstream analyses. Three independent co-culture experiments were performed, each in duplicate [24].

### 2.11. Western Blot Analysis

To obtain cell lysates, following 24 h of incubation, HepG2 cells from co-culture experiments were scraped into 50 μL of ice-cold lysis buffer (RIPA buffer + inhibitor cocktail + 1:100 PMSF + 1:100 sodium orthovanadate) and transferred to ice-chilled microcentrifuge tubes. Liver tissues were homogenized in the same buffer on ice using 2.0 mm zirconium beads for 4 min at maximum speed (BeadBug™ homogenizer, Benchmark Scientific, Sayreville, NJ, USA). Samples were centrifuged at 16,060× *g* for 15 min at 4 °C, and supernatants were collected into fresh pre-chilled tubes. Total protein concentrations were determined by the Bradford method, and 50 μg of protein per sample was loaded onto precast 7.5% SDS-PAGE gels and run at 130 V for 45 min. Proteins were transferred to nitrocellulose membranes (Mini Nitrocellulose Transfer Packs, Bio-Rad) using a Trans-Blot Turbo (1.3 A, 25 V, and 7 min, Bio-Rad). After blocking with milk or BSA, target proteins were probed with the following primary antibodies: anti-SREBP2, anti-LDLR, anti-HMGCoAR, anti-phospho-HMGCoAR (Ser872), anti-pAMPK (Thr172), anti-pAkt (S473), anti-GLUT 4, anti-PPAR-γ, anti-FASN, and anti-β-actin. HRP-conjugated secondary antibodies and chemiluminescent detection were used for visualization, and signals were quantified with Image Lab software version 3.01 (Bio-Rad, Hercules, CA, USA). β-Actin served as an internal control to normalize loading variability.

### 2.12. Fluorescent LDL Uptake Cell-Based Assay

HepG2 cells (3 × 10^4^ cells/well) were seeded in 96-well plates and maintained in complete growth medium for 2 days prior to treatment. On day 3, cells were treated with 67 × 10^6^ ALVs/mL or vehicle (100 mM Tris) for 24 h. For experiments, HepG2 cells were exposed to 67 × 10^6^ ALVs/mL in DMEM lacking FBS for 24 h. At the end of treatments, the medium was replaced with 75 μL/well of LDL-DyLight549 working solution. After an additional 2 h at 37 °C, the medium was aspirated and replaced with 100 μL/well PBS. LDL uptake was quantified on a Synergy H1 fluorescent plate reader (excitation/emission of 540/570 nm, Agilent, Santa Clara, CA, USA).

### 2.13. Fluorescent Glucose Uptake Cell-Based Assay

HepG2 cells (3 × 10^4^/well) were seeded into 96-well plates and kept in complete growth medium for 2 days. On day 3, cells were washed once with PBS and treated with 67 × 10^6^ ALVs/mL or vehicle (water) in DMEM without FBS for 24 h. The next day, the medium was removed and 75 μL/well of 2-NBDG (100 μg/mL) in DMEM without FBS was added for 10 min at 37 °C. Excess 2-NBDG was carefully aspirated without disturbing the HepG2 monolayer, and cells were washed twice with 100 μL of cell-based assay buffer. Glucose uptake was then measured on a Synergy H1 fluorescent plate reader (excitation/emission of 485/535 nm).

### 2.14. Oil Red O Staining

HepG2 cells (3 × 10^4^) were seeded in 96-well plates and kept in complete growth medium for 24 h. After 24 h, HepG2 cells were treated with 20.4 × 10^6^ ALVs/mL for 24 h. To induce lipid accumulation, the day after, HepG2 cells were treated with 1 mM oleate/palmitate mixture (OP). After the incubation time, the medium was removed, HepG2 cells were washed three times with PBS to remove unbound staining, and then fixed with 10% formalin for 1 h. After washing three times with distilled water, cells were washed with 60% isopropanol briefly and incubated with 60% filtered Oil Red O staining for 1 h to stain the adipocytes. Then, the staining was removed and cells were washed twice with PBS. Since the Oil Red O staining solution was used to stain the adipocytes, absorbance at 490–520 nm was measured using the Synergy H1 absorbance plate reader from Biotek.

### 2.15. In Vivo Hypoglycemic Assessment

A total of 20 4-week-old C57BL/6 male mice from the University of Seville Animal Facility were divided into four groups: mice fed with a standard diet (SD, *n* = 4); SD + ALVs (*n* = 5); high-fat and high-fructose diet (HFHF, *n* = 5); and HFHF + ALVs (*n* = 6). SD was the control group for SD + ALVs, while HFHF was the control group for HFHF + ALVs. The HFHF diet composition is reported in the Supplementary Materials (Table S1). Animals were intragastrically treated daily with physiological saline solution or ALVs for 12 weeks (5 days/week), and individual body weight was measured and recorded weekly. The mice were kept and housed under standard and pathogen-free conditions (12/12 light/dark cycles, temperature of 22 ± 2 °C, and humidity <55%) with ad libitum water and food at the Instituto de Biomedicina de Sevilla (IBiS). The impact of the ALVs on the mice’s glycemic response was assessed through an oral glucose tolerance test (OGTT), as described previously [25]. Briefly, fasted mice were intragastrically treated with saline or ALVs, and after 30 min, the blood glucose concentration was quantified by vein tail puncture using the Accu-Chek^®^ instant glucometer (Roche Diagnostic, Bassel, Switzerland) and an oral glucose load (2 g/kg) was administered. The levels of blood glucose were measured at intervals of 15, 30, and 60 min following oral glucose intake. The area under the curve (AUC) was calculated for each animal using the trapezoid rule. Once the OGTT had been carried out, the mice were sacrificed. All experimental procedures were conducted under the Spanish legislation and EU Directive 2010/63/EU for animal experiments and were approved by the Ethics Committee of the Virgen Macarena and Virgen del Rocío University Hospitals (reference 06/07/2023/60).

### 2.16. Assessment of Biochemical Parameters on Mice Plasma and Liver Tissue

Plasma parameters were assessed using chemiluminescence immunoassay methods with the COBAS e601 modular analyzer (Roche Diagnostic, Basel, Switzerland). In addition, the cardiovascular disease risk index, CRI-II, was calculated according to Kalelioglu and colleagues [26]. On the other hand, 100 mg of liver tissue was homogenized using a TissueRuptor (Qiagen, Hilden, Germany), and hepatic markers were analyzed in the supernatants using the Cobas Integra 400 (Roche Diagnostics, Indianapolis, IN, USA) at the ‘Estación Biológica de Doñana’ (EBD-CSIC, Seville, Spain).

### 2.17. Statistical Analysis

All data sets were analyzed using one-way ANOVA after verifying assumptions with Brown–Forsythe and Bartlett’s tests for homogeneity of variances, followed by Tukey’s post hoc analysis (GraphPad Software 9, San Diego, CA, USA). Values were expressed as means ± standard deviation; *p*-values ≤ 0.05 were considered significant.

## 3. Results

### 3.1. ALVs Show Antioxidant Properties and Modulate In Vitro Cholesterol, Glucose, and Lipid Metabolisms

**Figure 1:**
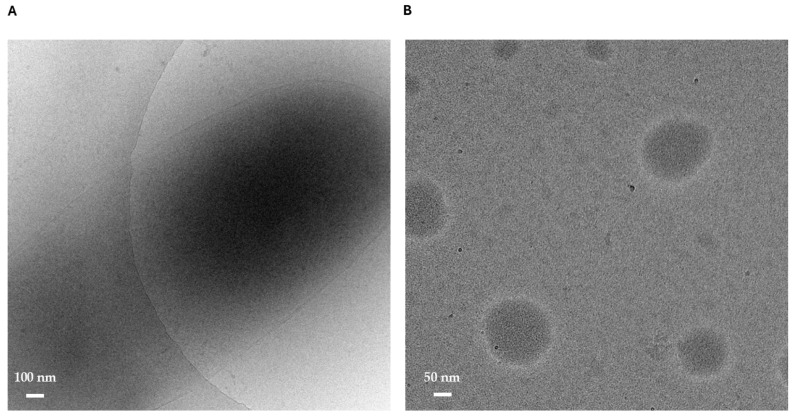
Cryogenic electron microscopy of ALVs. (**A**) Structures measuring 0.5–2 μm with a sharp, membrane-like boundary and a densely filled interior (scale bar: 100 nm). (**B**) Smaller 50–200 nm structures lacking a defined edge, consistent with lipidic droplets or nanoemulsion-like particles (scale bar: 50 nm).

Arugula leaf vesicles were obtained according to the previously patented method [15]. More in detail, nanoparticle tracking analysis (NTA) previously showed that a stable ALV population with a median diameter (D50) of 122 ± 2 nm was obtained with a yield of 68 × 10^6^ vesicles/mg, and exhibited a narrow nanoscale size distribution without detectable aggregates. Dynamic light scattering (DLS) reported a hydrodynamic diameter of 131 ± 11 nm with a PDI of 0.431, indicating good sample homogeneity. Zeta potential measurements indicated a negative surface charge (approximately –24 to –28 mV), consistent with good colloidal stability [16]. Cryogenic electron microscopy (cryo-EM), as shown in Figure 1A,B, further confirmed ALV integrity, revealing well-defined membrane-bound structures ranging from 0.5 to 2 μm, as well as smaller 50–200 nm particles consistent with lipidic droplets. Overall, these results demonstrated that the obtained ALVs are homogeneous, structurally preserved, and suitable for subsequent biochemical investigations.

**Figure 2:**
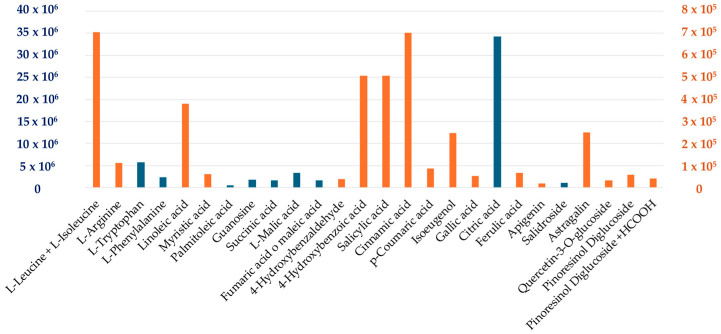
ALVs metabolite relative abundance. The ALV cargo composition was analyzed using Liquid Chromatography–High-Resolution Mass Spectrometry (LC-HRMS). A total of 26 metabolites were identified, including amino acids, fatty acids, polyphenols, carboxylic acids, and purine bases.

Given the composition of ALVs, rich in amino acids, fatty acids, polyphenols, and organic acids [16] (Figure 2), the first aim of the study was to investigate their antioxidant properties in vitro on cellular models.

**Figure 3:**
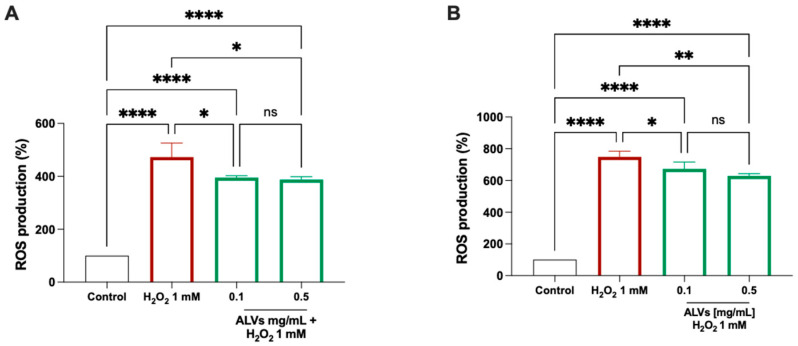
Antioxidant effects of ALVs on Caco-2 and HepG2 cells. ROS production in Caco-2 (**A**) and HepG2 (**B**) treated with 0.1 and 0.5 mg/mL ALVs stimulated with 1 mM H_2_O_2_. Grey lines refer to control cells. Red lines refer to the ROS production induced by H_2_O_2_ alone and green lines refer to the ROS production in cells treated with ALVs 0.1 and 0.5 mg/mL in presence of H_2_O_2_. Data represent the mean ± s.d. of six independent experiments performed in triplicate. All data sets were analyzed by one-way ANOVA. ns: not significant; (*): *p* < 0.5; (**): *p* ≤ 0.01; (****): *p* < 0.0001. Control: untreated cells; 0.1 mg/mL ALVs = 6.7 × 10^6^ ALVs/mL; 0.5 mg/mL ALVs = 33.5 × 10^6^ ALVs/mL.

Figure 3 shows that the treatment of Caco-2 cells and HepG2 with H_2_O_2_ alone produces a significant increase in intracellular ROS levels up to 472.6 ± 53.33% and 748.9 ± 35.78%, which was attenuated by the pretreatment with 0.1 and 0.5 mg/mL ALVs, which significantly reduced the ROS levels up to 395 ± 7.10% and 387.8 ± 10.82%, respectively, in Caco-2 cells, and 672 ± 43.34% and 629.2 ± 13.79% in HepG2, respectively. These findings indicate that both ALV concentrations significantly protected the Caco-2 cells and HepG2 cells from the H_2_O_2_-induced oxidative stress.

**Figure 4:**
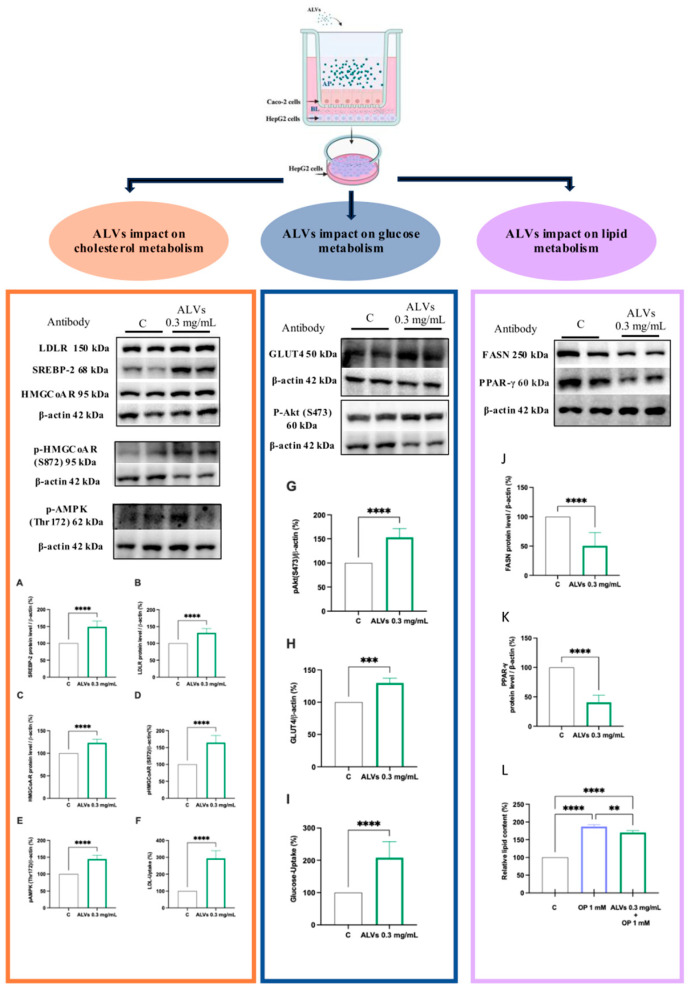
Schematic representation of Caco-2/HepG2 co-culture system and the effect of absorbed ALVs on cholesterol, glucose, and lipid metabolisms, respectively, in HepG2 cells. Caco-2 cells underwent a 21-day growth and differentiation process on polycarbonate filter membranes. Simultaneously, confluent human hepatic HepG2 cells were seeded in 12-well culture plates with complete medium. During co-culture experiments, filter inserts with differentiated Caco-2 cells were moved to wells containing hepatic cell cultures. Caco-2 cells were treated with 1 mg/mL ALVs (67 × 10^6^ ALVs/mL) for 24 h in the apical chamber. After the ALVs treatment, HepG2 cells in contact with the adsorbed ALVs (~30%) in the co-culture system were harvested and processed for immunoblotting experiments. The following molecular targets were investigated to study the cholesterol metabolism: SREBP-2 (**A**), LDLR (**B**), HMGCoAR (**C**), pHMGCoAR (Ser872) (**D**), pAMPK (Thr172) (**E**), the glucose metabolism pAkt (S473) (**G**), and GLUT4 (**H**), and the lipid metabolism: FASN (**J**) and PPAR-γ (**K**) in HepG2 cells. pHMGCoAR (Ser872) and pAMPK (Thr172) were probed on the same nitrocellulose membrane; therefore, both targets were normalized to the same β-actin loading control, which explains why the corresponding β-actin blot images appear identical (**D**,**E**). According to these molecular results, the LDL uptake (**F**), the glucose uptake (**I**), and the Oil Red O staining (**L**) experiments clearly show ALV treatments induce an increase in the ability to uptake LDL and glucose from the extracellular environment, and the reduction in the lipid accumulation in HepG2 cells. The terms AP (apical side) and BL (basolateral side) indicate the respective model chambers. Bars represent averages of duplicate-sample SEMs of three independent experiments. Grey lines refer to the control cells, green lines refer to cells treated with ALVs, violet lines refer to the cells pretreated with O/P + ALVs. All data sets were analyzed by one-way ANOVA followed by Tukey’s post hoc test. (****): *p* ≤ 0.0001; (***): *p* ≤ 0.001; (**): *p* ≤ 0.01. Control: untreated cells. ALVs: arugula leaf nanovesicles 0.3 mg/mL = 20.4 × 10^6^ ALVs/mL.

We previously demonstrated that nearly 30% of intact ALVs can be uptaken by differentiated Caco-2 cells and cross the basolateral membrane, being intact in the BL solution, thus demonstrating the intestinal bioavailability of ALVs [16]. To investigate the absorbed metabolic impact of ALVs, a co-culture system was set up by combining intestinal Caco-2 cells and hepatic HepG2 cells to mimic in vitro the physiological crosstalk existing between the intestine and liver. Caco-2 cells were differentiated on filter inserts and hepatic HepG2 cells were grown at the bottom of the culture plates (Figure 4). The treatment of intestinal cells with ALVs (67 × 10^6^ ALVs/mL or 1 mg/mL) induced an upregulation of the protein level of the SREBP-2 N-terminal fragment (the mature form with a molecular weight of 68 kDa) in the underlying HepG2 cells co-cultured up to 131 ± 2.95% (Figure 4A). Consequently, ALVs increased both the LDLR and HMGCoAR protein levels up to 143.01 ± 6.029% and 123.24 ± 2.832%, respectively (Figure 4B,C). Lastly, by increasing the pAMPK (Thr172) protein levels up to 144.63 ± 4.447, ALVs increased the p-HMGCoAR up to 164.51 ± 6.761 (Figure 4D,E). Thus, ALVs increased the capacity of HepG2 cells to uptake the LDL from the extracellular environment up to 293.5 ± 13.15% (Figure 4F). In addition, ALVs activated the Akt pathway by significantly increasing its phosphorylation level up to 153.05 ± 7.430% (Figure 4G). In agreement with the Akt and AMPK pathway activation, ALVs increased the GLUT4 protein level up to 129.78 ± 3.660% (Figure 4H), which led to an increased HepG2 ability to uptake extracellular glucose (2-NBDG) up to 208 ± 17.54% (Figure 4I). Moreover, ALVs decreased both fatty acid synthase (FASN) and peroxisome proliferator-activated receptor γ (PPAR-γ) levels up to 49.59 ± 6.24% and 59.48 ± 4.62%, respectively (Figure 4J,K), showing, from a functional point of view, a reduction in lipid accumulation in HepG2 cells induced by the oleate/palmitate mix (OP). Notably, the 1 mM OP treatment significantly increased the relative lipid content (%) up to 187 ± 5.462%, and the ALV pretreatment reduced the lipid accumulation up to 170.2 ± 5.776% (Figure 4L and Figure S3).

### 3.2. In Vivo Characterization of ALVs

#### ALVs Impact on Metabolic Health in HFHF Diet-Fed Mice Ameliorating the Glycemic Response and Modulating the Metabolic and Hepatotoxic Biomarkers

**Figure 5:**
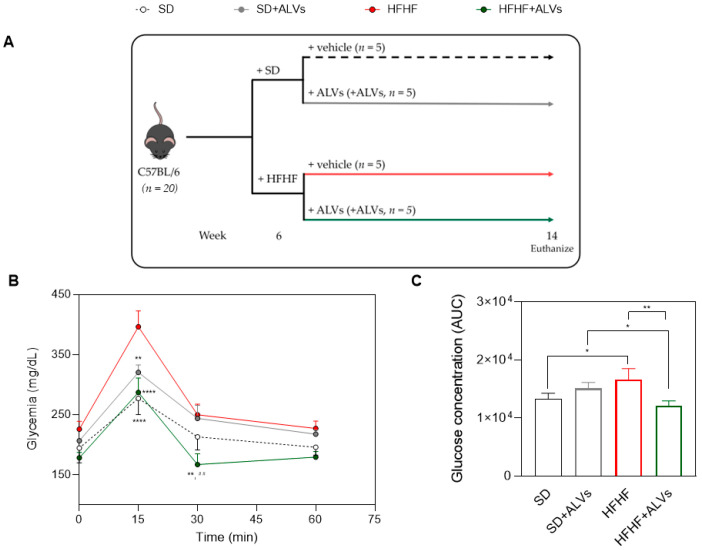
In vivo experiment results. (**A**) Study design scheme. (**B**) Oral glucose tolerance test (OGTT) in mice fed with SD or HFHF and treated with saline or ALVs. After 30 min of the treatments, blood glucose (time 0) was measured and a glucose load was immediately administered. Then, 15, 30, and 60 min after the glucose load, the blood glucose concentration was measured again. (**C**) Area under the curve calculated from the glycemia curve. Data were represented as mean ± standard deviation. For (**B**): **: *p* ≤ 0.01; ****: *p* ≤ 0.0001 with respect to the HFHF group; ##: *p* ≤ 0.01 with respect to the SD + ALV group. ALVs, arugula leaf vesicles; HFHF, high-fat and high-fructose diet; SD, standard diet. For (**C**): *: *p* ≤ 0.05; **: *p* ≤ 0.01.

Fifteen minutes after glucose ingestion, the animals reached peak glycemia levels (Figure 5B). The HFHF group reached higher glucose concentrations (396.25 ± 52.65 mg/dL), whereas HFHF + ALV-treated mice (287.00 ± 47.70 mg/dL) showed glucose levels comparable to those of the SD (227 ± 26.94 mg/dL) and SD + ALV (320.25 ± 24.58 mg/dL) groups. Notably, the average increase in glucose concentration at the peak of the curve was 38% greater in the HFHF control group compared to the ALV-treated group. Moreover, the ALV treatments induced a rapid return to normal glucose levels, with concentrations reaching 167.00 ± 35.68 mg/dL al 30 min.

In Figure 5C, the HFHF group also showed higher AUC values (16,662 ± 1849) than the SD (13,343 ± 951) or SD + ALV (15,100 ± 1032) groups. In contrast, the HFHF + ALV group showed a substantial reduction in the AUC (12,088 ± 847), representing a significant improvement compared to the HFHF group (*p* = 0.0004), and restoring glucose homeostasis to normal levels

**Figure 6:**
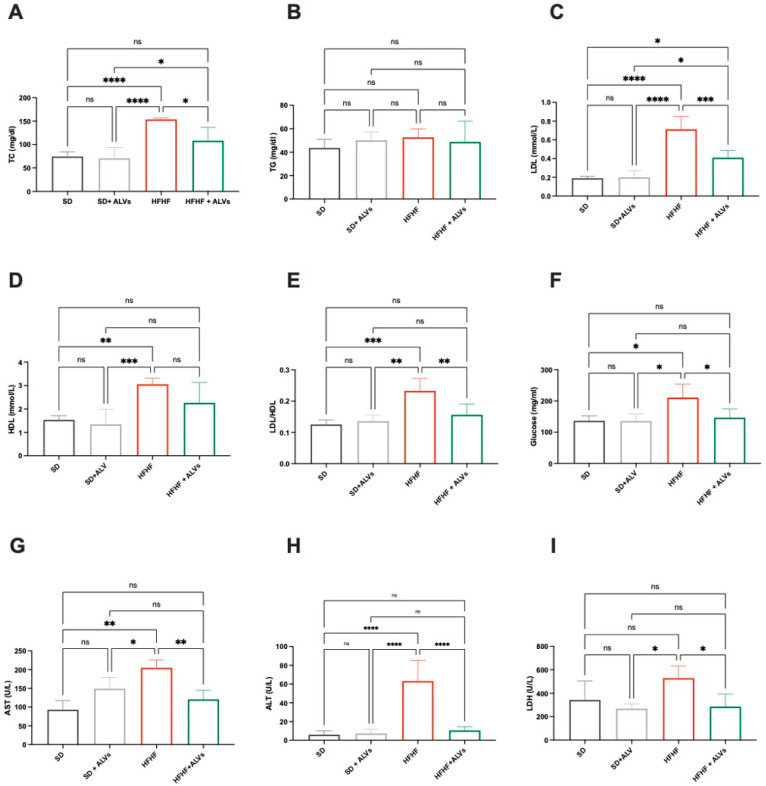
Metabolic parameters and liver injury biomarkers of C57BL/6 mice serum fed with SD, SD + ALVs, HFHF diet, and HFHF + ALVs. (**A**) Total cholesterol, (**B**) TG, (**C**) LDL, (**D**) HDL, (**E**) LDL/HDL, (**F**) glucose, (**G**) AST, (**H**) ALT, and (**I**) LDH were measured in the animal sera. Dark grey lines refer to animal fed with SD, light grey lines refer to animal fed with SD+ALVs, red lines refer to animal fed with HFHF diet, green lines refer to animal fed with HFHF diet+ALVs. ns: not significant; (*): *p* ≤ 0.05; (**): *p* ≤ 0.01; (***): *p* ≤ 0.001; (****): *p* ≤ 0.0001. TC: total cholesterol; TG: triglyceride; LDL: low-density lipoprotein; HDL: high-density lipoprotein; AST: aspartate aminotransferase, ALT: alanine transaminase, LDH: lactate dehydrogenase.

Biochemical analysis (Figure 6A–I) of mice plasma revealed that an HFHF diet worsened the metabolic and hepatotoxic biomarker profiles in blood plasma compared to the SD. This was highlighted by increased levels of total cholesterol (up to 153.5 ± 3.2 mg/mL), low-density lipoprotein (LDL) (up to 0.71 ± 0.3 mmol/L), LDL/HDL (Castelli Risk index II, 0.23 ± 0.03), glucose (up to 210.1 ± 43.25 mg/mL), aspartate aminotransferase (AST) (up to 204.6 ± 21.95 U/L), alanine transaminase (ALT) (up to 63.16 ± 21.96 U/L), and lactate dehydrogenase (LDH) (up to 528.4 ± 104.5 U/L) (Figure 6). Conversely, the HFHF + ALV group showed clear reductions in these biomarkers: total cholesterol (108.1 ± 28.92 mg/dL), LDL (up to 0.41 ± 0.077 mmol/L), LDL/HDL (up to 0.1558 ± 0.03514), glucose (up to 145.8 ± 28.77 mg/mL), AST (up to 120.4 ± 25.30 U/L), and ALT and LDH (up to 10.47 ± 3.96 U/L and 285.2 ± 107.7 U/L, respectively) (Figure 6A–I). Furthermore, ALV supplementation did not affect these parameters in SD-fed mice, indicating a favorable safety profile. This suggests that ALV administration does not negatively modulate serum toxicity markers, further supporting their potential exploitation.

**Figure 7:**
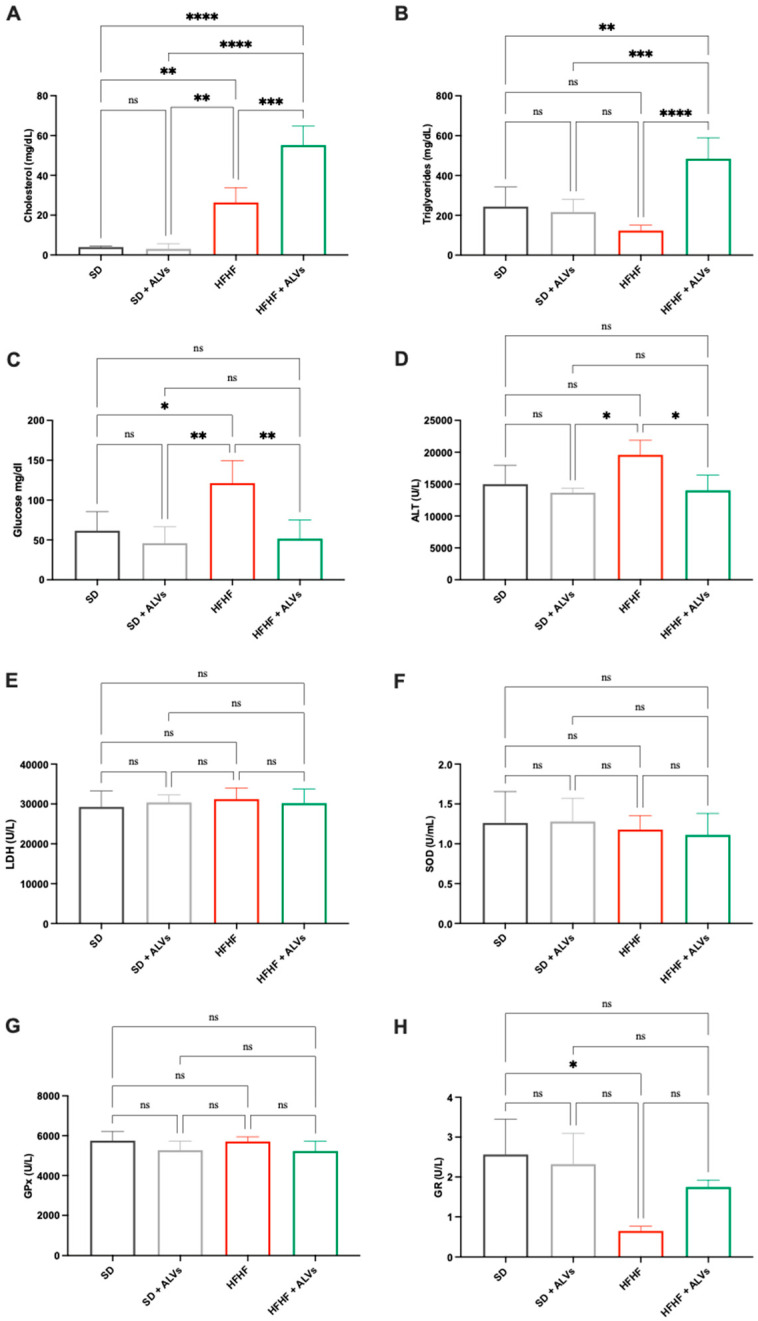
Metabolic and liver injury biomarkers concentration in liver homogenates of C57BL/6 mice fed with SD, SD + ALVs, HFHF diet, HFHF + ALVs. Dark grey lines refer to animal fed with SD, light grey lines refer to animal fed with SD + ALVs, red lines refer to animal fed with HFHF diet, green lines refer to animal fed with HFHF diet + ALVs. (**A**) Cholesterol, (**B**) triglycerides, (**C**) glucose, (**D**) ALT, (**E**) LDH, (**F**) SOD, (**G**) GPx, and (**H**) GR were measured in the animals’ livers. ns: not significant; (*): *p* ≤ 0.05; (**): *p* ≤ 0.01; (***): *p* ≤ 0.001; (****): *p* ≤ 0.0001. ALT: alanine transaminase; LDH: lactate dehydrogenase; SOD: superoxide dismutase; GPx: glutathione peroxidase; GP: glutathione reductase.

Given the liver’s crucial role in glucose and lipid metabolisms, as well as detoxification, key metabolic and hepatotoxic biomarkers were analyzed in liver homogenates of the experimental groups (Figure 7). Biochemical analysis showed that, compared to SD-fed mice, the HFHF diet increased liver glucose levels up to 121.3 ± 28.28 mg/dL, while the ALV administration in HFHF-fed mice reduced glucose levels up to 51.65 ± 23.41 mg/dL (Figure 7A). No differences in triglyceride concentrations were observed between SD and HFHF groups (Figure 7B). However, triglyceride levels increased significantly up to 483.9 ± 105.1 mg/dL in the HFHF + ALV group. Regarding total cholesterol, the HFHF diet and HFHF + ALV group showed increases up to 26.37 ± 11.10 and 55.21 ± 9.5 mg/dL, respectively, compared to the SD group. To investigate the potential hepatotoxicity of ALVs, key enzymes indicative of liver toxicity (ALT and LDH) and oxidative stress (GPx, GR, and SOD) were quantified in liver homogenates (Figure 7D–H). The results (Figure 7E–G) showed no significant modulation of LDH, SOD, or GPx among SD, SD + ALVs, HFHF, and HFHF + ALVs, indicating that ALVs do not induce hepatotoxicity. Notably, ALT (Figure 7D) levels were higher in livers from HFHF-fed mice (up to 19,585 ± 2309 U/L), but significantly decreased in HFHF + ALV-fed mice (up to 14,019 ± 2372 U/L). Similarly, GR levels decreased (Figure 7H) in HFHF-fed mice (0.653 ± 0.11 U/L) but increased significantly in the HFHF + ALV group (1.44 ± 0.55 U/L). These findings suggest the safety profile of ALVs, which is further supported by the absence of the negative modulation of hepatotoxicity and oxidative stress markers in the livers of SD + ALV-fed mice.

**Figure 8:**
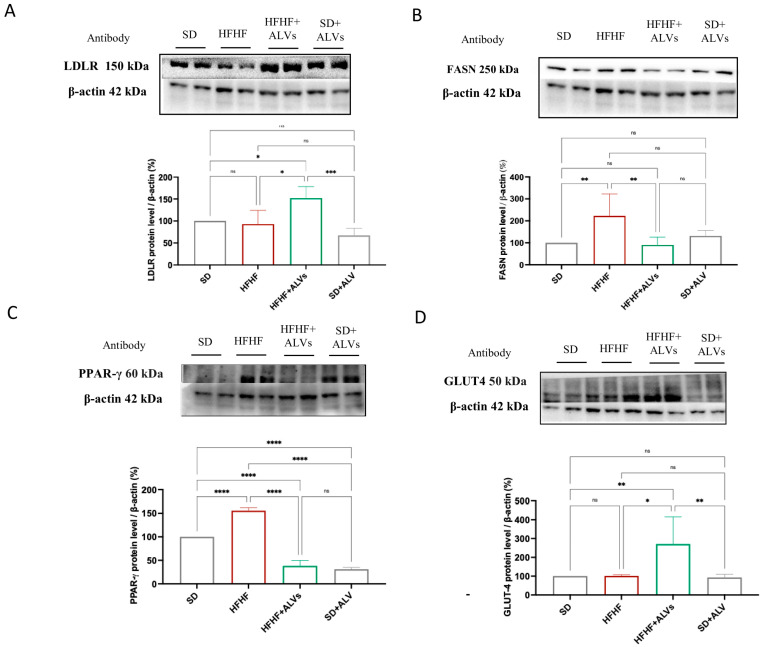
ALV impact on main targets involved in cholesterol metabolism, glucose, and lipid metabolism in mice livers. The following molecular targets were investigated to study the cholesterol, lipid, and glucose metabolisms in liver homogenates: LDLR (**A**), FASN (**B**), PPAR-γ (**C**), and (**D**) GLUT4 in SD, SD + ALV, HFHF, and HFHF + ALVs fed mice. LDLR and FASN were probed on the same nitrocellulose membrane; therefore, both targets were normalized to the same β-actin loading control, which explains why the corresponding β-actin blot images appear identical (**A**,**B**). All data sets were analyzed by one-way ANOVA followed by Tukey’s post hoc test. ns: not significant; (****): *p* ≤ 0.0001; (***): *p* ≤ 0.001; (**): *p* ≤ 0.01; (*): *p* ≤ 0.5.

LDLR, FASN, PPAR-γ, and GLUT4 proteins were quantified in the livers of the mice of the four experimental groups to investigate the effect of ALV supplementation on lipid, cholesterol, and glucose metabolisms. Figure 8 shows that, while no significant changes in LDLR and GLUT-4 protein levels were observed between the SD and HFHF groups, the HFHF + ALV group showed an increase in the LDLR and GLUT-4 protein levels up to 152.2 ± 26.05% and 270.9 ± 144.3% compared to the control, respectively. In the HFHF group, an increase in FASN and PPAR-γ protein levels were observed up to 222.9 ± 99.76% and 155.8 ± 6.10%, respectively, compared to the SD group. Interestingly, the HFHF + ALV group showed a decrease in the FASN and PPAR-γ protein levels up to 90.87 ± 34.50% and 38.23 ± 11.54%, respectively.

## 4. Discussion

PDVs are nanoscale structures that represent a rapidly expanding area because of their natural capacity to carry and deliver bioactives, demonstrating several biological properties [27,28]. While several studies suggests that PDVs from different plant sources may exhibit antioxidant activity in cellular and animal models, the mechanism of action remains poorly investigated [29]. As a matter of fact, several studies have demonstrated the immunomodulatory, microbiota-regulating, antioxidant, and anti-aging activities of PDVs, owing to their content of bioactive components, since these nanovesicles are bioformulated structures that encapsulate and deliver bioactive compounds from the source plant, preserving their integrity and enhancing their biological efficacy [30]. Literature evidence shows that plant nanovesicles exhibit multi-organ therapeutic potential, protecting the brain, skin, heart, gut, liver, and bone by crossing biological barriers, modulating inflammation, oxidative stress, and promoting regeneration and differentiation across diverse tissues [31,32,33]. Focusing on ready-to-eat arugula, which has a short shelf life and high environmental impact when discarded, ALVs were previously isolated using a previously developed and patented protocol (WO 2024/223549 A1) designed to preserve the morphological and physicochemical properties of plant nanovesicles. ALVs were successfully produced and stabilized, obtaining 67 × 10^6^ ALVs/mg, not aggregated, stable at room temperature, having a negative charge, and a diameter between 87 and 214 nm. LC-HRMS analysis identified 26 secondary metabolites belonging to amino acids (e.g., leucine/isoleucine, arginine, and tryptophan), fatty acids (linoleic, myristic, and palmitoleic), carboxylic and phenolic acids (e.g., succinic, malic, salicylic, citric, and ferulic), polyphenols (salidroside), flavonoids (apigenin, astragalin, and quercetin-3-O-glucoside), and furanoid lignans (pinoresinol derivatives). This profile highlights the diverse bioactive cargo composition of ALVs, which is linked to the biological activity and that has been characterized by using both in vitro and in vivo studies. Furthermore, we demonstrated that ALVs can be efficiently internalized by differentiated Caco-2 cells, demonstrating their ability to be uptaken intact by intestinal cells, and to cross the intestinal cell basolateral membrane to reach the basolateral Transwell system compartment intact. Interestingly, given the highly heterogeneous composition of ALVs, regarding not only secondary metabolites but also plant miRNAs, our findings align with the multifunctional activity observed, which extends beyond the antioxidant effects. Importantly, compared to traditional extracts and phytochemicals, the strengths of PDVs are related to their low toxicity, their ability to protect their cargo from degradative phenomena, and their capacity to be uptaken at the cellular level. Glucose and lipid metabolisms are central to energy balance and overall health, and the impairment of these processes can lead to metabolic disorders, such as obesity, type 2 diabetes, nonalcoholic fatty liver disease, and cardiovascular diseases, which are increasingly prevalent worldwide [34]. Hepatic oxidative stress is closely linked to disturbances in cholesterol and the glucose and lipid metabolisms, which are hallmarks of metabolic diseases such as type 2 diabetes, obesity, and non-alcoholic fatty liver disease. Excess ROS generated in the liver can impair mitochondrial and endoplasmic reticulum function, leading to altered lipid synthesis and oxidation, increased cholesterol accumulation, and insulin resistance. These alterations promote hepatic steatosis and dyslipidemia, further enhancing ROS production and inflammation [35,36,37]. In light of these aspects, we first investigated the antioxidant properties of ALVs at the cellular level. Our findings demonstrate that ALVs at the lowest concentration tested, 0.1 mg/mL, show antioxidant properties at the cellular level on Caco-2 and HepG2 cells (Figure 3). To further explore the biological properties of ALVs, we developed a co-culture model consisting of differentiated human intestinal Caco-2 cells and hepatic HepG2 cells cultured in the basolateral compartment to mimic in vitro the physiological crosstalk between the intestine and the liver. This system was designed to investigate the beneficial effects of bio-absorbed ALVs at the hepatic level and to elucidate their molecular mechanisms of action, which were subsequently evaluated in an in vivo *proof-of-concept* study. Importantly, our findings suggest that, once transported across the intestinal epithelium, ALVs positively modulated the LDLR pathway by increasing receptor protein levels through SREBP-2 activation (see Figure 4A). In agreement with this result, an increased level of HMGCoAR was observed (Figure 4C). Notably, HMGCoAR activity can be modulated by a reversible phosphorylation–dephosphorylation, with the phosphorylated form of the enzyme being inactive (70%) and the dephosphorylated form being active (30%) [38]. Thus, when HMGCoAR is phosphorylated, the synthesis of de novo cholesterol is reduced. This short-term phosphorylation of HMGCoAR is induced by pAMPK, which deactivates HMGCoAR via phosphorylation on Ser872. In this regard, statins can activate (phosphorylate) AMPK, inhibiting HMGCoAR. In this study, an increase in the phosphorylated form of HMGCoAR and AMPK in ALV-treated cells, compared to the control HepG2 cells (Figure 4D,E), was observed, suggesting that ALV acts on HMGCoAR activity through AMPK phosphorylation. From a functional perspective, the activation of the LDLR pathway resulted in the enhanced uptake of LDL by hepatic cells from the extracellular environment, ultimately leading to an in vitro hypocholesterolemic effect (Figure 4F).

Interestingly, ALVs did not directly inhibit HMGCoAR, suggesting a mechanism of action which is different from statins [39], and providing the ability of ALVs to enter cells, where they can modulate the cholesterol metabolism. To confirm this hypothesis, ALVs were sonicated to alter their structural integrity. When tested with the HepG2 cells, LDLR modulation was achieved; when structurally intact, their ability to increase LDLR was maintained (Figure S2). Moreover, the trans-epithelial transport of ALVs successfully activated the Akt pathway, increasing its phosphorylation of Ser473 levels (Figure 4G), leading to an improvement of GLUT4 levels in HepG2 cells, which improved the functional ability of hepatic cells to uptake extracellular glucose with a hypoglycemic effect (Figure 4I). In light of these results, and considering the physiological crosstalk between Akt and AMPK pathway activations in the glucose and lipid metabolisms, the trans-epithelial-transported ALVs impact the lipid metabolism [40,41] through the reduction in FASN protein levels, suggesting a reduction in de novo lipogenesis. It is well known that the accumulation of PPARγ in the liver induces hepatic steatosis through the activation of lipogenic genes by increasing hepatic lipogenesis and triglyceride synthesis [42]. Hence, in agreement with this result, a reduction in PPARγ protein levels was detected (Figure 4K), leading, from a functional point of view, to a reduction in the OP-induced lipid content in intracellular HepG2 (Figure 4L and Figure S3). Taking all of these results together, it appears clear that ALVs, which can efficiently cross differentiated Caco-2 cells, suppress liver gluconeogenesis and lipid and cholesterol production, while decreasing hepatic lipid deposition via AMPK and Akt modulation, thus improving glucose and lipid profiles in HepG2 cells. Subsequently, to further extend our in vitro observations, we evaluated the metabolic impact of ALV supplementation in a murine model in an in vivo study. The antidiabetic potential of ALVs was demonstrated in vivo, providing the *proof of concept* of their efficacy. Results (Figure 5) suggested that ALVs succeeded in controlling glucose level regulation in a metabolic syndrome mice model, where mice were fed a high-fat and high-fructose diet (HFHF). Subsequent biochemical analyses of plasma and liver homogenates indicate that, while the HFHF diet leads to a general worsening of plasma metabolic markers, the administration of ALVs in the HFHF group ameliorates the metabolic profile. Specifically, while the HFHF diet leads to increased levels of plasma total cholesterol, LDL, and glucose concentration, as well as Castelli Risk index II (LDL/HDL), these parameters are significantly reduced in the HFHF + ALV group (Figure 6A,C,E,F). Concerning the biochemical analysis conducted on liver homogenates, the HFHF + ALV diet reduces the glucose concentration, while increasing the cholesterol and triglyceride concentration (Figure 7).

Based on these results, Western blotting analyses were performed on liver homogenates to further investigate the link between the modulation of plasma lipid parameters and the effects of ALVs on the hepatic cholesterol metabolism pathway. Our findings demonstrate that HFHF + ALV mice show increased expression of LDLR and GLUT-4 protein levels, which would justify the accumulation of cholesterol and triglycerides in the livers, while reducing the protein levels of FASN and PPAR-γ (Figure 8). These results corroborate the in vitro data that suggest the ability of ALV to modulate both glucose and lipid metabolisms, respectively (Figure 4). Moreover, ALVs reduced the increase in the glucose concentration in an oral glucose tolerance test. ALVs not only can reduce the peak glucose concentration induced by the glucose load but also rapidly restore normal values. All of these findings related to the improvement of the plasma lipid profile, the enhancement of the glycemic response, along with the increased expression levels of the LDL receptor, the reduction in PPARγ, and the decreased FASN protein levels in the liver, corroborate the in vitro results associated with the hypocholesterolemic, hypoglycemic, and lipid-lowering effects of ALVs. Overall, these results clearly point out that these nano-phytocomplexes might be successfully exploited for the prevention of metabolic syndrome, which is a condition that includes a cluster of risk factors specific for cardiovascular disease in which oxidative stress occurs [35]. In line with our results, literature evidence demonstrates that nanovesicles obtained from blueberry could ameliorate oxidative stress in rotenone-induced HepG2 cells and high-fat diet (HFD)-fed C57BL/6 mice by improving mitochondrial function, activating Nrf2 signaling, and increasing antioxidant protein expression; in HFD-fed mice, these nanovesicles also improved insulin resistance, restored liver function, and inhibited lipid accumulation [43]. Interestingly, a 2020 study demonstrates that nanovesicles derived from orange juice show biological properties by reversing some gut alterations caused by a high-fat and high-sugar diet in obese mice. These vesicles improved the intestinal structure, reduced triglyceride accumulation in the gut, and modulated genes involved in inflammation and lipid metabolism. However, they did not improve insulin resistance or blood lipid levels after one month of treatment [44].

Remarkably, nanovesicles derived from plants are inherently highly biocompatible, as they are already present in commonly consumed foods, and there is growing evidence supporting that they could have the potential to exert positive effects on human health [27,45]. Importantly, compared to traditional extracts and phytochemicals, the strengths of PDVs are related to their low toxicity, their ability to protect their cargo from degradative phenomena, and their capacity to be uptaken at the cellular level. In this scenario, in the present study, we proposed a tiered approach, starting from cellular models and shifting to the *proof-of-concept* in vivo study, to obtain a comprehensive safety profile of ALVs. Data from MTT experiments carried out at different concentrations, both on human intestinal Caco-2 and hepatic HepG2 cell lines, confirmed that ALVs did not cause any toxic effect either on human intestinal cells or on human hepatic cells, indicating a favorable in vitro safety profile of the nano-phytocomplex. Our findings are in line with recent studies suggesting that plant-derived nanovesicles obtained from a variety of sources, such as cabbage, blueberries, strawberries, grapefruit, bitter melon, carrots, and ginger, showed no toxicity at the cellular level [46,47]. Owing to their natural origin and good biocompatibility, plant-derived nanovesicles have attracted considerable attention; indeed, several in vivo studies have been conducted with plant-derived nanovesicles demonstrating their numerous health-promoting effects [48,49]. This is besides showing in vivo that they possess good biological safety and do not exhibit significant toxic effects on major organs, even though their safety aspects have not been comprehensively explored in depth. During our in vivo studies carried out in mice models, for mice intragastrically administered with ALVs, no signs of systemic toxicity was present, as there was no significant increase in serum AST, ALT, or LDH levels in mice fed with SD compared to those given SD + ALVs. Additionally, homogenates from the livers of mice in the SD + ALV group did not exhibit a significant increase in ALT, LDH, and oxidative stress markers (GPx, GR, and SOD). The same trend was noticed for HFHF + ALV-fed mice, demonstrating that ALV supplementation in standard and dysmetabolic conditions did not alter normal mice physiology. These results are in line with the generally safe profile of plant-derived nanovesicles, being bio-formulated bioactive cargo with high biocompatibility.

## 5. Conclusions

This study demonstrates that ALVs represent an innovation in food supplements and the nutraceutical filed, being a novel opportunity for the development of plant-derived, advanced nano-delivery systems of bioactive compounds synergically involved in carrying out different biological activities. In addition, clear evidence has been provided highlighting the unique multifunctional biological activity of ALVs, which are able to modulate glucose, cholesterol, and lipid metabolisms, respectively, in both in vitro and in vivo models. However, even though ALVs show a great potential for further exploitation in the prevention of metabolic syndrome, the present study is limited by the lack of additional information needed to fully characterize their functional properties. Indeed, a dedicated clinical study would be essential to further reinforce and validate these promising in vitro and in vivo findings.

## 6. Patents

The above method, which includes ALV isolation, purification, and stabilization, is referenced in the patent WO 2024/223549 A1, “Method for the production of plant-derived nanovesicles and their applications”. It has been licensed to Plantech s.r.l.

## Data Availability

The original contributions presented in this study are included in the article/Supplementary Materials. Further inquiries can be directed to the corresponding author.

## Supplementary Materials

The following supporting information can be downloaded at: https://www.mdpi.com/article/10.3390/antiox14121421/s1, Figure S1: Evaluation of ALV effects on cellular viability; Figure S2: Effect of lysed ALVs and ALVs on LDLR in HepG2 cells; Figure S3: Representative image of Oil Red O staining on HepG2 cells; Table S1: HFHF diet composition.

## Abbreviations

The following abbreviations are used in this manuscript:

2-NBDG	2-(N-(7-Nitrobenz-2-oxa-1,3-diazol-4-yl)Amino)-2-Deoxyglucose
Akt	Protein Kinase B
ALT	Alanine Transaminase
ALVs	Arugula Leaf Vesicles
AMPK	AMP-Activated Protein Kinase
ANOVA	Analysis of Variance
AST	Aspartate Aminotransferase
AUC	Area Under the Curve
BSA	Bovine Serum Albumin
CRI-II	Castelli Risk Index II (LDL/HDL ratio)
Cryo-EM	Cryogenic electron microscopy
DLS	Dynamic Light Scattering
DMEM	Dulbecco’s Modified Eagle Medium
DMSO	Dimethyl Sulfoxide
FASN	Fatty Acid Synthase
FBS	Fetal Bovine Serum
GLUT4	Glucose Transporter Type 4
GPx	Glutathione Peroxidase
GR	Glutathione Reductase
H_2_O_2_	Hydrogen Peroxide
HDL	High-Density Lipoprotein
HFHF	High-Fat and High-Fructose
HFHF + ALVs	High-Fat and High-Fructose + Arugula Leaf Vesicles
HMGCoAR	3-Hydroxy-3-Methylglutaryl-CoA Reductase
LDH	Lactate Dehydrogenase
LDL	Low-Density Lipoprotein
LDLR	Low-Density Lipoprotein Receptor
miRNA	microRNA
MTT	3-(4,5-Dimethylthiazol-2-yl)-2,5-Diphenyltetrazolium Bromide
NTA	Nanoparticle Tracking Analysis
OGTT	Oral Glucose Tolerance Test
OP	Oleate/Palmitate Mixture
p-HMGCoAR	Phosphorylated HMG-CoA Reductase
pAkt (Ser473)	Phosphorylated Akt at Serine 473
pAMPK (Thr172)	Phosphorylated AMPK at Threonine 172
PBS	Phosphate Buffered Saline
PDVs	Plant-Derived Vesicles
PMSF	Phenylmethylsulfonyl Fluoride
PPAR-γ	Peroxisome Proliferator-Activated Receptor Gamma
RIPA buffer	Radioimmunoprecipitation Assay Buffer
ROS	Reactive Oxygen Species
SD	Standard Diet
SD + ALVs	Standard Diet + Arugula Leaf Vesicles
SDS-PAGE	Sodium Dodecyl Sulfate–Polyacrylamide Gel Electrophoresis
SEM	Standard Error of the Mean
SOD	Superoxide Dismutase
SREBP-2	Sterol Regulatory Element-Binding Protein 2
TC	Total Cholesterol
TEER	Transepithelial Electrical Resistance
TG	Triglyceride
β-actin	Beta-actin

## Appendix A. ALV Chemical Composition Analysis: Secondary Metabolite Determination by HR-HPLC-MS/MS

Polyphenol extraction was performed in triplicate, and the entire procedure was carried out in darkness. ALV powder was homogenized in acidified methanol (0.1% *v*/*v* HCl) at 4% *w*/*v*. The mixture was then placed in an ultrasonic bath for 15 min and centrifuged at 12,500 rpm for 5 min. The pellet was discarded, and the supernatant was stored at –80 °C until analysis. Samples were analyzed at UNITECH OMICs (University of Milan, Italy) using the ExionLC™ AD system (SCIEX) coupled to the ZenoTOF 7600 System (SCIEX), equipped with a Turbo V™ Ion Source and ESI probe. Chromatographic separation was performed on a CORTECS UPLC T3 column (1.6 µm, 2.1 × 150 mm; Waters™, Milford, MA, USA) using mobile phase A (0.1% formic acid in water) and mobile phase B (0.1% formic acid in acetonitrile) at a flow rate of 400 µL/min. The elution program is summarized in Table A1. Column and autosampler temperatures were maintained at 40 °C and 10 °C, respectively, and the injection volume was 5 µL. MS spectra were acquired in negative-ion mode over an *m*/*z* range of 50–1500 Da using IDA^®^ (Information Dependent Acquisition). Collision energy was set at 30 (CES 15). Data processing was performed with SCIEX OS v3.0™ software integrated with LibraryView v1.4, which includes the Natural Products HR-MS/MS Library (over 1000 spectra), and Formula Finder, an algorithm that predicts candidate chemical formulae based on the precursor ion mass accuracy and isotopic pattern. Analytes were identified by comparing experimental MS/MS spectra with those in the consulted databases. A “non-targeted screening workflow” was also applied to detect additional analytes not included in the database through the assignment of putative molecular formulae.

**Table A1:** Elution program.

Time	Flow (µL/min)	%A	%B
0	400	99	1
2	400	99	1
6	400	75	25
10	400	5	95
15	400	5	95
15.20	400	99	1
20.00	400	Stop run	

**Table A2:** Parameters adopted for qualitative characterization.

Qualitative Rule	Green	Yellow	Red
Mass Error (ppm)	<5 ppm	<10 ppm	≥10 ppm
Library Hit Score	>70%	>50%	≤50%
Formula Finder Score	>50%	>20%	≤20%
