# Supplementary material for: Multifunctional Biological Activity Assessment of Plant-Derived Nanovesicles from Arugula Leaves: In Vitro and In Vivo Studies
Source: Antioxidants (Basel). 2025 Nov 27;14(12):1421. doi: 10.3390/antiox14121421 (PMC12730054; doi:10.3390/antiox14121421)
Supplement: Supplementary file 1 [file antioxidants-14-01421-s001.zip › antioxidants-3968505-supplementary.pdf]

Supplementary Figures and Tables

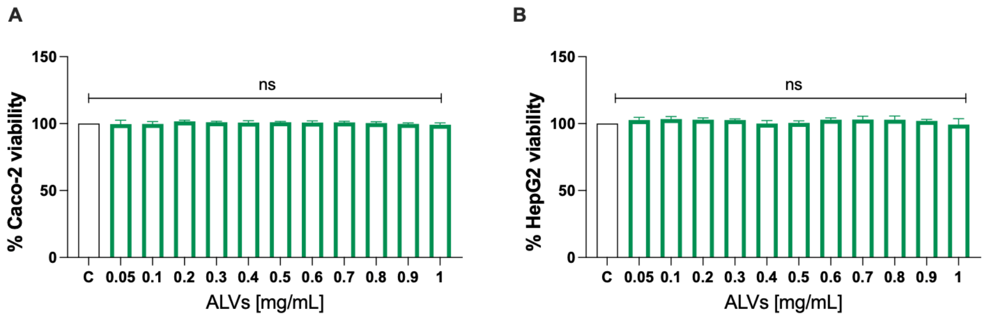

Figure S1. – Evaluation of ALVs effects on cellular viability. Effect of ALVs on the Caco-2 (A), HepG2 (B) cells viability. Data represents the mean ± s.d. of four independent experiments performed in duplicate. All data sets were analyzed by one-way ANOVA followed by Tukey's post hoc test. C: untreated cells. ns, not significant. ALVs [0.05 mg/mL] = 3,35 × 10^6 ALVs/mL. ALVs [0.1 mg/mL] = 6,7 × 10^6 ALVs/mL. ALVs [0.2 mg/mL] = 13,4 × 10^6 ALVs/mL. ALVs [0.3 mg/mL] = 20,1 × 10^6 ALVs/mL. ALVs [0.4 mg/mL] = 26,8 × 10^6 ALVs/mL. ALVs [0.5 mg/mL] = 33,5 × 10^6 ALVs/mL. ALVs [0.6 mg/mL] = 40,2 × 10^6 ALVs/mL. ALV [0.7 mg/mL] = 46,9 × 10^6 ALVs/mL. ALVs [0.8 mg/mL] = 53,6 × 10^6 ALVs/mL. ALVs [0.9 mg/mL] = 60,3 × 10^6 ALVs/mL. ALVs [1 mg/mL] = 67 × 10^6 ALVs/mL.

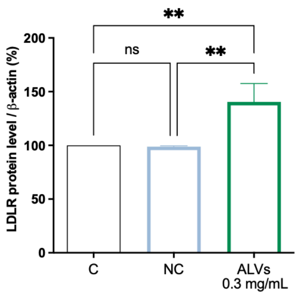

Figure S2. – Effect of lysed ALVs and ALVs on LDLR in HepG2 cells. ALVs structural integrity was damaged by sonication for few minutes. To study whereas the impact on cholesterol metabolism was kept after ALVs membrane damaging, HepG2 cells were treated with/or lysed ALVs (NC) and ALVs 0.3 mg/mL for 24 h. The day after, HepG2 cells were processed for immunoblotting experiments. LDLR and β-actin immunoblotting signals were detected using specific anti-LDLR and anti-β-actin primary antibodies, respectively. Each protein signal was quantified by ImageLab software (Biorad) and normalized with β-actin signals. Bars represent averages of duplicate samples SEM of three independent experiments. All data sets were analyzed by one-way ANOVA followed by Tukey's post hoc test. Control: untreated cells. NC: lysed ALVs; ALVs: arugula leaves nanovesicles 0.3 mg/mL = 20.1 × 10^6 ALVs/mL. (**) $p \leq 0.01$; ns: not significant.

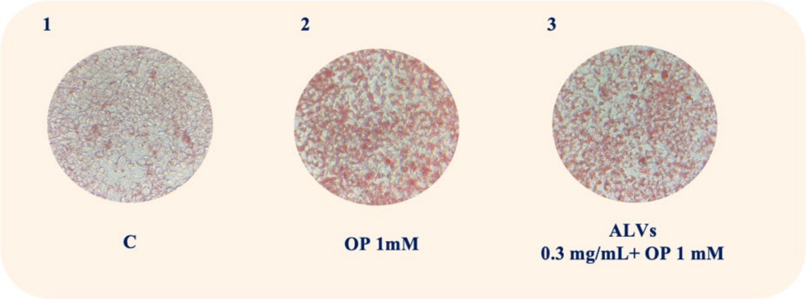

Figure S3. Representative image of Oil Red O staining on HepG2 cells. HepG2 cells were seeded 96-wells plate. The day after, HepG2 cells were pre-treated with ALVs 0.3 mg/mL. After 24 hours, HepG2 cells were treated with oleate/palmitate (OP) 1 mM for 24 h to induced lipid accumulation. After 24 hours, the pre-treatment with ALVs reduce the lipid accumulation in HepG2 cells compared to cells only treated with OP 1 mM. Observe the staining condition of each well after oil red O staining of adipogenesis induction under microscope. Oil red O staining was performed to compare the ability of adipogenesis. C: untreated cells (1), OP 1 mM cells treated with OP 1 mM (2), and cells pretreated with ALVs 0.3 mg/mL and OP 1 mM for 24 h (3).

Table S1: HFHF diet composition.

Altromin Spezialfutter GmbH & Co. KG
Im Seelenkamp 20 - D-32791 Lage
Tel.: +49 (0)5232/6088-0 - Fax: +49 (0)5232/6088-20
E-Mail: info@altromin.de - http://www.altromin.de

2.11.2021 19:09 Seite: 1

Nummer **Lfd. Nummer** Änderung 06.10.2021 17:39:02
10008805 108805 C1088-05 NASH high fructose + palm oil(w/40%fat, w/20%fructos, 2%chol)

Nr.	Inhaltsstoff	Einheit	Bedarf	Gehalt	Differenz
1	Rohprotein / Crude Protein	mg/kg		170782,922	
2	Rohfett / Crude Fat	mg/kg		220285,971	
3	Rohfaser / Crude Fibre	mg/kg		55382,504	
4	Rohasche / Crude Ash	mg/kg		54653,404	
5	Feuchtigkeit / Moisture	mg/kg		50681,246	
6	Monosaccharide(s)	mg/kg		200514,949	
7	Disaccharide(s)	mg/kg		7499,250	
8	Polysaccharide(s)	mg/kg		195270,473	
9	Umsetzb. Energie/Metab. Energy	kcal/kg		4350,650	
10	Lysin / Lysine	mg/kg		17340,381	
11	Methionin / Methionine	mg/kg		10647,435	
12	Cystin / Cystine	mg/kg		3153,995	
13	Threonin / Threonine	mg/kg		7078,807	
14	Tryptophan	mg/kg		1964,124	
15	Arginin / Arginine	mg/kg		9731,832	
16	Histidin / Histidine	mg/kg		5218,783	
17	Isoleucin / Isoleucine	mg/kg		7145,475	
18	Leucin / Leucine	mg/kg		14520,763	
19	Phenylalanin / Phenylalanine	mg/kg		7072,908	
20	Valin / Valine	mg/kg		3201,810	
21	Alanin / Alanine	mg/kg		2369,763	
22	Asparaginsäure / Aspartic acid	mg/kg		3449,285	
23	Glutaminsäure / Glutamic acid	mg/kg		23297,785	
24	Glycin / Glycine	mg/kg		3056,694	
25	Prolin / Proline	mg/kg		12577,652	
26	Serin / Serine	mg/kg		5164,584	
27	Tyrosin / Tyrosine	mg/kg		9198,375	
28	Vitamin A	I.E./kg		14998,500	
29	Vitamin D3	I.E./kg		499,950	
30	Vitamin E	mg/kg		150,385	
31	Vitamin K3 als/as Menadion(e)	mg/kg		9,999	
32	Vitamin B1	mg/kg		20,038	
33	Vitamin B2	mg/kg		20,320	
34	Vitamin B6	mg/kg		15,032	
35	Vitamin B12	mg/kg		0,041	
36	Nikotinsäure / Nicotinic acid	mg/kg		50,165	
37	Pantothensre./Pantothenic acid	mg/kg		50,101	
38	Folsäure / Folic acid	mg/kg		10,00140	
39	Biotin	mg/kg		0,201	
40	Cholinchlorid/Choline chloride	mg/kg		1011,399	
43	Inosit / Inositol	mg/kg		110,989	
44	Vitamin C	mg/kg		20,998	
45	Calcium	mg/kg		9003,870	
46	Ges.Phosphor / Phosphorus	mg/kg		7419,577	
47	Verd.Phosphor/Digest.Phosporus	mg/kg		7135,737	
48	Magnesium	mg/kg		663,354	
49	Natrium / Sodium	mg/kg		2419,622	
50	Kalium / Potassium	mg/kg		7020,346	
51	Schwefel / Sulfur	mg/kg		2742,749	
52	Chlor / Chlorine	mg/kg		3639,636	
